# Anti-N-Methyl-D-Aspartate Receptor Encephalitis With Diffuse Demyelinating Plaques: A Case Report of an Atypical Presentation

**DOI:** 10.7759/cureus.41595

**Published:** 2023-07-09

**Authors:** Luis-Angel Tirado-García, Steven-Andrés Piña-Ballantyne, Jesús Cienfuegos-Meza, Martha-Lilia Tena-Suck

**Affiliations:** 1 Neuropathology, Instituto Nacional de Neurologia y Neurocirugía Manuel Velasco Suárez, Mexico City, MEX; 2 Neuropathology, Instituto Nacional de Neurología y Neurocirugía Manuel Velasco Suárez, Mexico City, MEX

**Keywords:** anti-n-methyl-d-aspartate, anti-n-methyl-d-aspartate receptor, immunohistochemistry, gliosis, seizures, anti-nmdar encephalitis, autoimmune

## Abstract

Anti-N-methyl-D-aspartate (anti-NMDA) receptor encephalitis is an autoimmune disease triggered by antibodies against the NR1 subunit of this receptor. It has a wide variety of presentations, including abnormal behavior, psychosis, seizures, abnormal movement, insomnia, and irritability. The diagnosis is confirmed by the presence of one of the six main symptoms and anti-NR1 immunoglobulin G (IgG)-positive antibodies in the cerebrospinal fluid (CSF) after the exclusion of other disorders. We present a case of an 18-year-old female with progressive paresthesia and muscle weakness that compromised walking and psychiatric symptoms. She was admitted to a private institution where magnetic resonance imaging (MRI) revealed pseudotumoral lesions, which led to surgical intervention. The original histopathological diagnosis was of a pleomorphic xanthoastrocytoma (PXA) WHO grade 2. As symptoms persisted, she was referred to our institution where a new MRI was performed, and a biopsy was re-evaluated. It showed perivascular inflammatory infiltrates composed of T cells, intense peripheral gliosis, nodules of macrophages, and reactive astrocytes in the white matter with fragmentation and vacuolation of myelin sheets, suggesting a demyelinating process in contrast to neoplasia. CSF analysis was performed, and it was positive for anti-NMDA antibodies. Immunohistochemical positivity for N-methyl-D-aspartate (NMDA) was observed in the neuronal nuclei, which led to the diagnosis.

## Introduction

Autoimmune encephalitis (AE) is a group of inflammatory disorders of the brain characterized by antibodies against neuronal synaptic and cell surface antigens, causing synaptic receptor dysfunction. Its inflammatory pathogenesis produces a broad range of neurological symptoms, making diagnosis and clinical differentiation challenging. AE can be classified into two categories according to the location of their antigens: paraneoplastic encephalitis associated with antibodies targeting intracellular antigens and encephalitis associated with antibodies against neuronal cell surface or synaptic receptors [1].

The most common subtype is anti-N-methyl-D-aspartate receptor (anti-NMDAR) encephalitis, originally described in 2005 by Vitaliani et al. [2]. It is a subacute autoimmune neurological disorder with progressive psychosis, seizures, and autonomic dysfunction, leading to death if untreated [3]. It has an estimated incidence of one per 1.5 million cases a year, with female predominance (4:1 ratio), mostly between 25 and 35 years of age [4,5]. Anti-NMDAR is triggered by immunoglobulin G (IgG) antibodies against the NR1 subunit of the N-methyl-D-aspartate receptor (NMDAR) in the central nervous system [5], although immunopathogenesis of this disease remains obscure, B and T helper lymphocytes and macrophages have been proposed to be involved [6]. Clinically, anti-NMDAR encephalitis has a wide variety of presentations, mainly abnormal behavior, psychosis, seizures, abnormal movement, insomnia, and irritability [4,7].

Clinical features appear secondary to alterations in the structure and function of NMDAR caused by pathogenic antibodies against the epitopes of these proteins. These antibodies cause the internalization of NMDAR from synapses reducing neuronal calcium influx and decreasing synaptic currents; subsequently, the internalized receptors are destroyed in a proteasome-dependent manner [3,5].

Although rare, anti-NMDAR encephalitis can develop demyelinating lesions, causing overlapping syndromes that have been more frequently reported. Clinical characteristics among anti-NMDAR encephalitis with multiple sclerosis (MS), aquaporin-4-antibody-positive neuromyelitis optica spectrum disorder (AQP4-Ab-positive NMOSD), and myelin oligodendrocyte glycoprotein antibody-associated disease (MOGAD) are distinct [8]. Post-viral encephalitis, mainly due to herpes virus, and tumors are the main triggers for anti-NMDAR encephalitis [9,10].

In this report, we present a case of an 18-year-old female with progressive paresthesia and muscle weakness that compromised walking and psychiatric symptoms. Magnetic resonance imaging (MRI) showed heterogeneous lesions with ring artifacts that prompted a biopsy. MRI in this institution showed multiple demyelinated lesions in the white matter. Histopathological features, including secondary demyelinated lesions, are also presented.

## Case presentation

**Figure 1:**
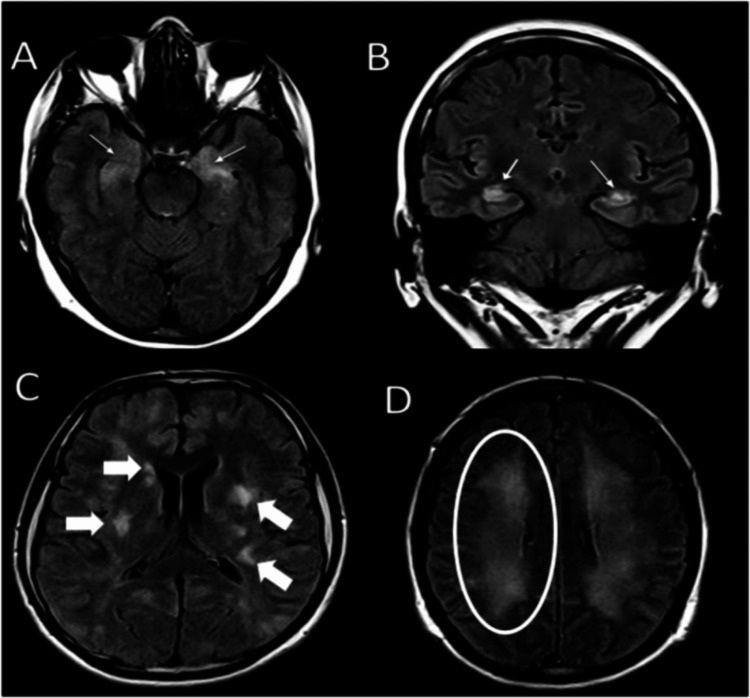
Cranial magnetic resonance imaging of hyperintense lesions MRI fluid-attenuated inversion recovery (FLAIR) sequence. (A and B) Bilateral hippocampal hyperintensities (light arrows). (C) Bilateral asymmetrical multifocal hyperintensities on cortical, subcortical, and periventricular zones (arrows). (D) Diffusive bilateral lesions on frontal lobes white matter (circle).

The patient was an 18-year-old woman with a family history of rheumatoid arthritis (mother) and major depressive disorder under unspecified treatment. She started three weeks before her first hospitalization with paresthesia of the left leg that progressed to muscle weakness with walking limitation. She attended a private institution where an MRI was performed, which showed multiple heterogeneous hyperintense lesions on T2 that were hypointense on T1; contrast medium manifested ring artifacts. This led to the diagnosis of a pseudotumoral lesion. A biopsy was performed with no complications. The original diagnosis was of a pleomorphic xanthoastrocytoma (PXA) WHO grade 2. After a week, the patient developed weakness in the contralateral limb, with a worsening psychiatric disorder; therefore, she was referred to our institute. A new MRI scan showed multiple irregular hyperintense lesions in the white matter of both hemispheres, with poor contrast enhancement and no edema. Both hippocampi were hyperintense and a similar lesion was also observed in the right mesencephalic peduncle (Figure 1).

On physical examination, the patient had to be placed in a wheelchair due to weakness in the lower extremities that progressed to the upper extremities, and a positive bilateral Babinski reflex was observed. She was hospitalized and received corticosteroid treatment with partial improvement (she was able to walk again). Two lumbar punctures were performed, and cerebrospinal fluid (CSF) analysis revealed no abnormalities. CSF analysis for anti-N-methyl-D-aspartate (anti-NMDA) antibodies was positive.

**Figure 2:**
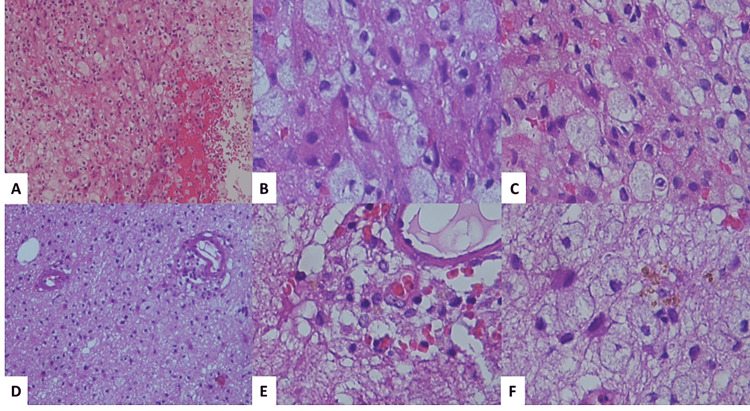
Hematoxylin & eosin (H&E) stain of paraffin block sample A diffuse infiltrate is formed by macrophages with vacuolated cytoplasm and localized hemorrhage (A-C). The neuropil acquired a microcystic appearance with a perivascular chronic inflammatory response formed by lymphocytes (D and E) and reactive astrocytes and hemosiderin (F) (H&E x400).

**Figure 3:**
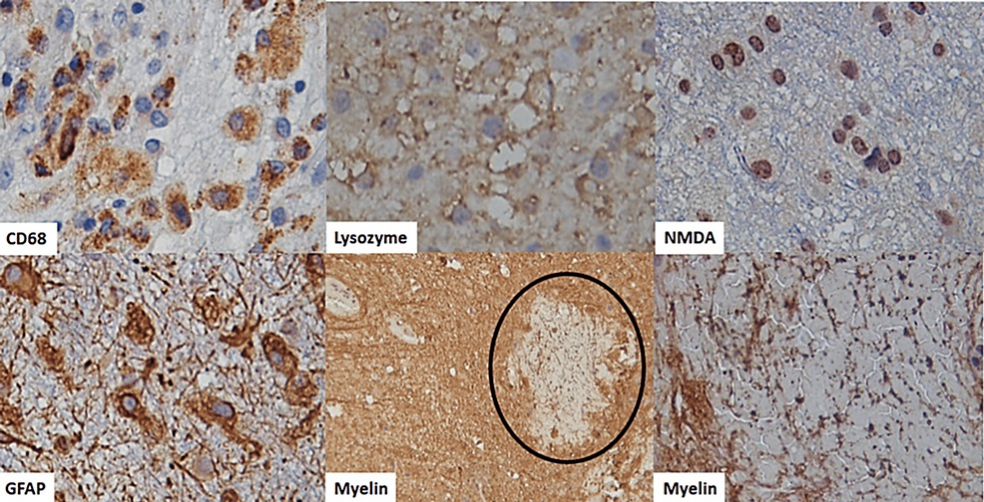
Immunohistochemistry of paraffin block sample Cluster of differentiation (CD68): positivity shows the presence of rich macrophage infiltration. Lysozyme: negative staining. N-methyl-D-aspartate (NMDA): strong nuclear staining on neurons. Glial fibrillary acidic protein (GFAP): positive for astrocytes with no atypical morphology. Myelin: demyelinating plaque (black circle) and demyelination with nonspecific pattern.

Re-analysis of the biopsy by two (neuro)pathologists (MLTS and JCM) showed immunohistochemistry positivity for N-methyl-D-aspartate (NMDA) in neuronal nuclei. No neuronal loss was evident. Mild perivascular inflammatory infiltrates were composed of clusters of differentiation three and four (CD3+ and CD4+) cells. Glial fibrillary acidic protein (GFAP) showed intense peripheral gliosis and highlighted gemistocytes. Additionally, nodules of macrophages and reactive astrocytes in the white matter demonstrated fragmentation and vacuolation of myelin sheets (Figure 2). This suggested a demyelinating process. Markers for tubulin and neurofilaments also showed fragmented axonal processes. Unfortunately, antibodies for demyelinating disease were not performed (Figure 3). Intravenous immunoglobulin was administered with good clinical improvement and recovery of symptoms. The patient was discharged after significant improvement.

## Discussion

In this clinical case, anti-NMDAR encephalitis was clinically diagnosed at our institution, aided by CSF-positive anti-NMDA antibodies and histopathological features. A biopsy was performed in a previous institution, due to, perhaps, a differential diagnosis of lymphoma. Moreover, the first histopathological diagnosis was PXA, a glial tumor with very specific features. This highlights the complex assessment of this disease in non-specialized hospitals.

The clinical course of this patient was not typical, as she developed generalized weakness and an inability to walk. MRI features showed extensive white matter lesions not explained by anti-NMDAR encephalitis. Besides hyperintensities in both hippocampi, frontoparietal subcortical and periventricular white matter were found. Biopsy showed that these lesions belong to a demyelinating process. In cases with both anti-NMDAR encephalitis and demyelinating disease, so-called overlapping syndrome, MRI abnormalities are described as having increased multifocal subcortical lesions involving subcortical, periventricular, deep white matter, as well as infratentorial regions such as the spinal cord, compared to cortical hyperintensities showed in anti-NMDAR encephalitis alone [10].

Anti-NMDAR encephalitis is diagnosed clinically by the recognition of six main symptoms, and prodromal symptoms are also described [7]. The diagnosis is confirmed in the presence of one of the six main symptoms and anti-NR1 IgG-positive antibodies after the exclusion of other disorders [10].

Immunopathogenesis in anti-NMDAR encephalitis is not well understood; however, multiple immune cell interactions mediated by cytokines/chemokines and T cells play an important role [6]. In patients with ovarian teratomas, or after herpes simplex encephalitis, it is proposed that the antigen against NMDA is generated in naïve B-cells that later cross the blood-brain barrier with the cooperation of CD4+ cells. Once in the brain parenchyma, these B-cells become activated and undergo further stimulation, antigen-driven maturation, and clonal expansion to produce anti-NMDA antibodies [11]. However, this proposed mechanism has not been fully proven.

In another study, Liba et al. [12] measured CSF levels of chemokines such as CXCL10 to attract T cells and CXCL13 to attract B cells, but also levels of cytokines associated with Th function such as interferon-gamma (IFN-γ), tumor necrosis factor-alpha (TNF-α), interleukin (IL)-4, and IL-17. They found that CXCL13 concentrations increased during the first two months but CXCL10 concentrations remained elevated for six months. The cytokine levels were elevated throughout the course of the disease. In this context, Liu et al. [13] emphasized the possibility of immunopathogenesis, T cells, and B cells, with T cells likely to assist B cells in antibody production during the acute phase. IL-7 promotes the differentiation of precursor B cells and precursor T cells. Th1 and Th2 axis-related cytokines/chemokines promote the differentiation of CD4+ T cells into Th1 and Th2 cells. CXCL10 and CCL3 mediate the chemotaxis of Th1 cells to pathological sites, whereas CCL1, CCL8, CCL17, and CCL22 mediate the chemotaxis of Th2 cells to pathological sites and stimulate the production of antibodies associated with B cells. CXCL10 and CCL3 mediate the chemotaxis of Th1 cells to pathologic sites, while CCL1, CCL8, CCL17, and CCL22 mediate the chemotaxis of Th2 cells to pathologic sites and stimulate the production of associated antibodies by B cells. Additionally, CXCL10 and CXCL13 can help B cells produce antibodies, and IL-6 is essential for B cells to differentiate into plasma cells and produce antibodies. These mechanisms may explain the histological findings of B cells in the absence of T cells since CSF analysis and biopsy were performed posterior to the patient's immunization.

Although brain biopsy is not the gold standard for diagnosis, it can be performed when the clinical presentation is not typical and there is considerable doubt about the disease [7,14]. Histopathological diagnosis is challenging because brain parenchyma shows a range of inflammation changes, including a few inflammatory cells and perivascular cuffs of T-cells, B-cells, and plasma cells. Neuronal loss is very mild, only seen in 25% of patients. Other findings are microglial activation, gliosis, and neuronal IgG binding to NMDARs without complement activation and deposition [2,14]. Data have shown reduced NMDAR immunohistochemical reactivity in the hippocampi, due to receptor internalization [15].

In our case, neocortical neurons' nuclei, white matter axonal processes, and oligodendrocytes showed NMDAR immunohistochemical positivity. This immunoexpression can be related to the demyelinated nodules with loss of myelin and macrophages. The precise mechanism of demyelination is not well understood. Patterns of demyelination have been described in tissues of patients with anti-NMDAR encephalitis and demyelinating lesions, suggesting a close link with multiple sclerosis [14]. White matter lesions are also described in anti-NMDAR encephalitis overlapping with neuromyelitis optica spectrum disorders (NMOSD9), acute demyelinating encephalomyelitis (ADE), and myelin oligodendrocyte glycoprotein (MOG) antibody-associated disease (MOGAD). Distinct clinical features have been described in each of these overlap syndromes [8].

NMDAR-AE is often responsive to immunotherapy with a good outcome if diagnosed and treated early. The prognosis varies depending on the treatment regimen, the need for surgical intervention, and the severity of the disease [16].

This case showed histopathological features of anti-NMDA encephalitis, although with mild cytotoxic inflammation of the parenchyma, with demyelinating nodules with loss of myelin and macrophages. Clinically, the patient improved after corticosteroid therapy alone, but testing for MS or other demyelinating disorders was not performed. If not suspected, clinical and radiological diagnosis can be challenging. In patients with overlapping syndrome, prompted clinical evaluation is necessary for proper treatment.

## Conclusions

Anti-NMDAR encephalitis has been the most studied autoimmune-mediated encephalitis in recent years; however, its pathophysiology remains to be elucidated. Diagnosis can be challenging, especially when an atypical presentation is observed. We presented the case of an 18-year-old woman with major depressive disorder in which a biopsy was obtained due to the diagnosis of a pseudotumor by MRI, originally diagnosed as PXA WHO grade 2 by histopathology. The biopsy was analyzed at our neuropathology department; a neoplastic lesion was discarded, and a demyelinating process was found. CSF analysis revealed anti-NMDA antibodies as well as immunohistochemistry, which, in relation to clinical symptoms, led to a conclusive diagnosis of anti-NMDAR encephalitis. Histopathological findings of anti-NMDAR encephalitis associated with demyelinating lesions are shown. This association must be considered clinically when patients develop motor dysfunction, in addition to the characteristic neuropsychiatric manifestations.

## References

[1] Kelley BP, Patel SC, Marin HL, Corrigan JJ, Mitsias PD, Griffith B (2017). Autoimmune encephalitis: pathophysiology and imaging review of an overlooked diagnosis. AJNR Am J Neuroradiol.

[2] Staley EM, Jamy R, Phan AQ, Figge DA, Pham HP (2019). N-methyl-D-aspartate receptor antibody encephalitis: a concise review of the disorder, diagnosis, and management. ACS Chem Neurosci.

[3] Lynch DR, Rattelle A, Dong YN, Roslin K, Gleichman AJ, Panzer JA (2018). Anti-NMDA receptor encephalitis: clinical features and basic mechanisms. Adv Pharmacol.

[4] Dalmau J, Armangué T, Planagumà J (2019). An update on anti-NMDA receptor encephalitis for neurologists and psychiatrists: mechanisms and models. Lancet Neurol.

[5] Samanta D, Lui F (2022). Anti-NMDA Receptor Encephalitis. https://www.ncbi.nlm.nih.gov/books/NBK551672/.

[6] Wagnon I, Hélie P, Bardou I (2020). Autoimmune encephalitis mediated by B-cell response against N-methyl-d-aspartate receptor. Brain.

[7] Graus F, Titulaer MJ, Balu R (2016). A clinical approach to diagnosis of autoimmune encephalitis. Lancet Neurol.

[8] Zhang S, Yang Y, Liu W, Li Z, Li J, Zhou D (2022). Clinical characteristics of anti-N-methyl-D-aspartate receptor encephalitis overlapping with demyelinating diseases: a review. Front Immunol.

[9] Liu P, Yan H, Li H, Zhang C, Li Y (2023). Overlapping anti-NMDAR encephalitis and multiple sclerosis: a case report and literature review. Front Immunol.

[10] Huang Q, Xie Y, Hu Z, Tang X (2020). Anti-N-methyl-D-aspartate receptor encephalitis: a review of pathogenic mechanisms, treatment, prognosis. Brain Res.

[11] Dalmau J (2016). NMDA receptor encephalitis and other antibody-mediated disorders of the synapse: the 2016 Cotzias lecture. Neurology.

[12] Liba Z, Kayserova J, Elisak M (2016). Anti-N-methyl-D-aspartate receptor encephalitis: the clinical course in light of the chemokine and cytokine levels in cerebrospinal fluid. J Neuroinflammation.

[13] Liu J, Liu L, Kang W (2020). Cytokines/chemokines: potential biomarkers for non-paraneoplastic anti-N-methyl-D-aspartate receptor encephalitis. Front Neurol.

[14] Zrzavy T, Endmayr V, Bauer J (2021). Neuropathological variability within a spectrum of NMDAR-encephalitis. Ann Neurol.

[15] Dalmau J, Tüzün E, Wu HY (2007). Paraneoplastic anti-N-methyl-D-aspartate receptor encephalitis associated with ovarian teratoma. Ann Neurol.

[16] Iizuka T, Kanazawa N, Yanagida A (2021). Anti-NMDA receptor encephalitis. (Article in Japanese). Brain Nerve.

